# Supplementary figures and images for: Social isolation but not deprivation involved in employment status after bariatric surgery
Source: PLoS One. 2021 Sep 10;16(9):e0256952. doi: 10.1371/journal.pone.0256952 (PMC8432780; doi:10.1371/journal.pone.0256952)

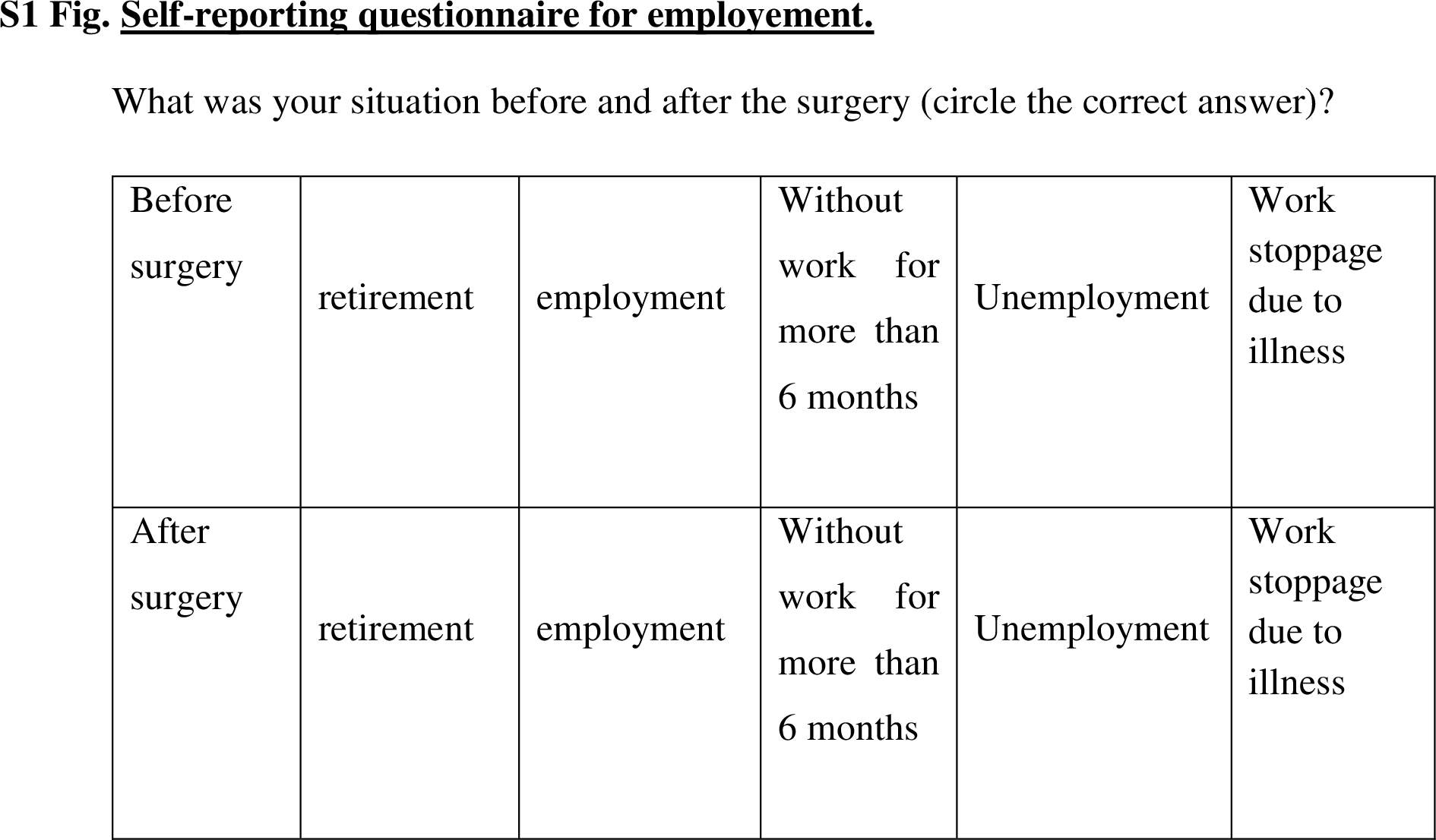

Supplement: S1 Fig — What was your situation before and after the surgery (circle the correct answer)?. (TIF) [file pone.0256952.s001.tif]

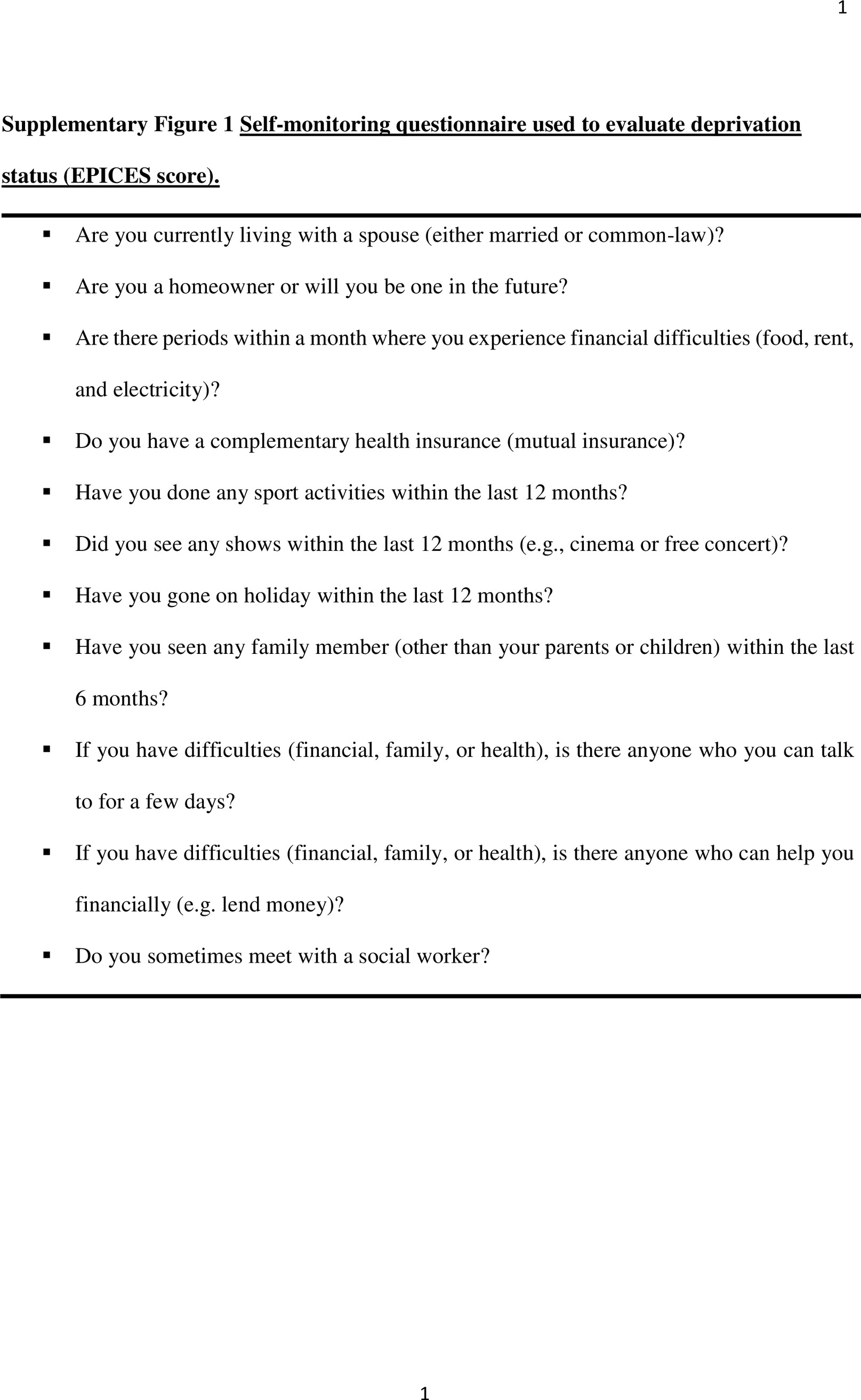

Supplement: S2 Fig — (TIF) [file pone.0256952.s002.tif]

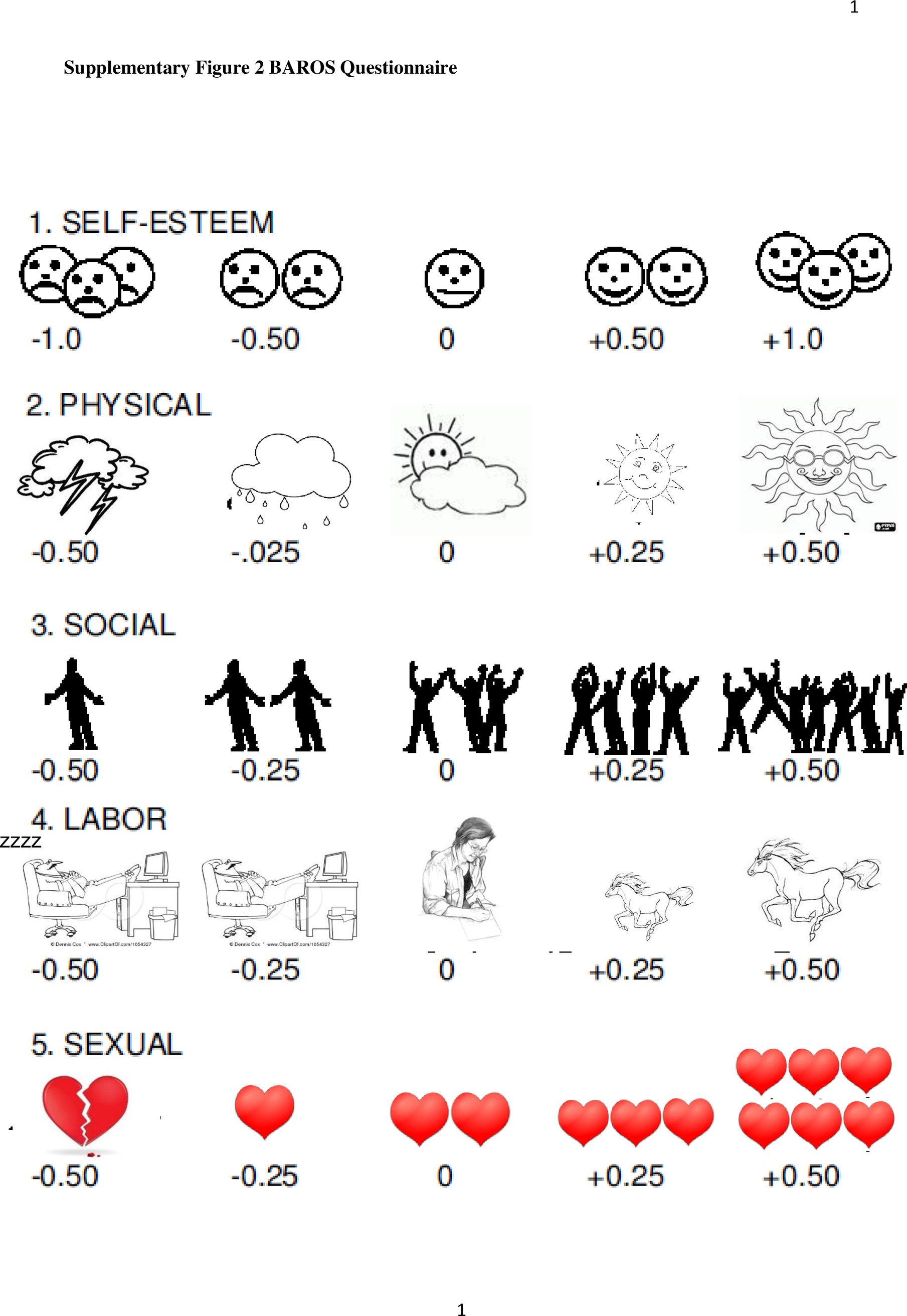

Supplement: S3 Fig — Satisfaction scale. What is your degree of satisfaction with bariatric surgery?. Satisfaction scale. What is your degree of satisfaction with bariatric surgery?. (TIF) [file pone.0256952.s003.tif]

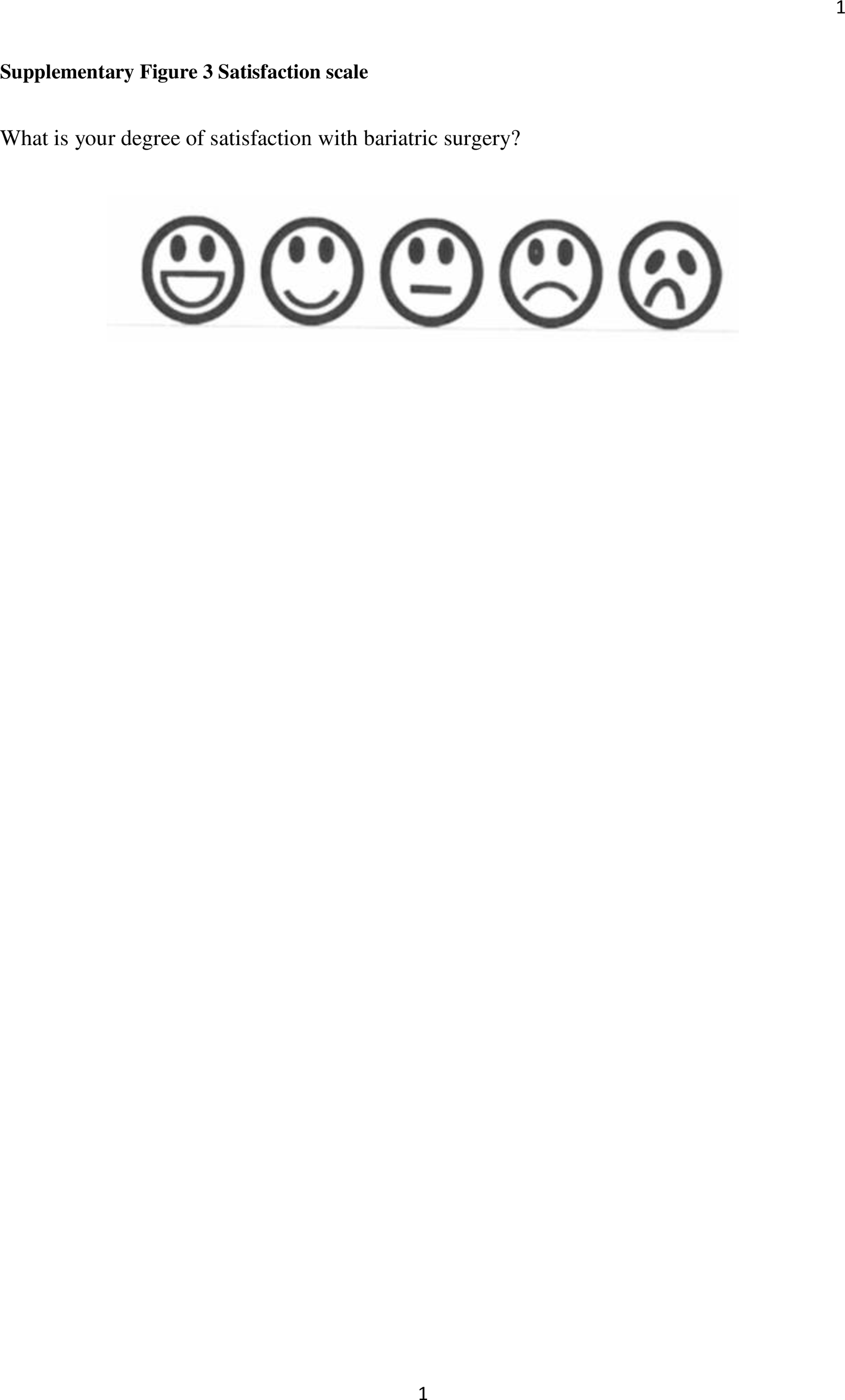

Supplement: S4 Fig — What is your degree of satisfaction with bariatric surgery?. (TIF) [file pone.0256952.s004.tif]
