# Supplementary material for: Social isolation but not deprivation involved in employment status after bariatric surgery
Source: PLoS One. 2021 Sep 10;16(9):e0256952. doi: 10.1371/journal.pone.0256952 (PMC8432780; doi:10.1371/journal.pone.0256952)
Supplement: Attachment — Submitted filename: Ethical response to reviewer.docx [file pone.0256952.s005.docx]

PONE-D-20-38829R1

Saint Denis the 9th of June 2021

Dear Editor

Pease find the answers to your request

1. Please amend your current ethics statement in the online submission form to include the full name of the ethics committee/institutional review board(s) that approved your specific study.

We added in material and method the following sentence Line 76 :

Ethics approval was granted by the local Saint Denis Hospital Ethics Committee and by the national Committee (Commission Nationale informatique et Liberté : ID number: 2220397 v 0 )

-       To help ensure that the wording of your manuscript is suitable for publication, would you please also add this statement at the beginning of the Methods section of your manuscript file.
2. We note your current data availability statement:

"The data are in an anonymous Excel file in my computer. I can send it on request"

PLOS only permits data to be made available on request if there are ethical or legal restrictions on sharing the data. Before we proceed, we’ll require some additional information to ensure your submission adheres to the PLOS policy on acceptable data access restrictions: [https://journals.plos.org/plosone/s/data-availability#loc-acceptable-data-access-restrictions](https://outlook.office.com/owa/redir.aspx?REF=Cl09xhFk-r0dL4BoRlZt4YMl3OGvOn8m9TJVtof0DIlqXyfjBivZCAFodHRwczovL2pvdXJuYWxzLnBsb3Mub3JnL3Bsb3NvbmUvcy9kYXRhLWF2YWlsYWJpbGl0eSNsb2MtYWNjZXB0YWJsZS1kYXRhLWFjY2Vzcy1yZXN0cmljdGlvbnM.).

a) Please confirm whether there are legal or ethical restrictions on sharing your data publicly.

b) If legal or ethical restrictions apply, please provide all necessary instructions and non-author contact information (preferably email) for a data access committee, ethics committee, or other institutional body other researchers would require to apply for data access. Note that it is not acceptable for an author to be the sole named individual responsible for ensuring data access.

c) If there are no legal or ethical restrictions on sharing your data publicly, please upload the minimal anonymized data set necessary to replicate your study findings as either Supporting Information files, or to a stable public repository. If you upload your data to a repository, please also provide the relevant URLs, DOIs, or accession numbers for other researchers to access your data directly. For a list of recommended public repositories, please see [https://journals.plos.org/plosone/s/recommended-repositories](https://outlook.office.com/owa/redir.aspx?REF=EwndsRq303Q05_dSzAU_0483bNL_xffc5FIIOgvLftVqXyfjBivZCAFodHRwczovL2pvdXJuYWxzLnBsb3Mub3JnL3Bsb3NvbmUvcy9yZWNvbW1lbmRlZC1yZXBvc2l0b3JpZXM.).

There is no ethical or legal restrictions on sharing our data. Our data do not contain potentially identifying or sensitive patient information. Anonymised Excel file was added to supplement material and available

<https://figshare.com/s/f7d57bd143b032429fee>

Once we receive this information, we will update your data availability statement on your behalf.

Your manuscript has been returned to your account. Please log on to PLOS Editorial Manager at [https://www.editorialmanager.com/pone/](https://outlook.office.com/owa/redir.aspx?REF=9oIoHIJRHCm7PTNktws9FXzbOGPfWtAL8pKZ-mWYLOlqXyfjBivZCAFodHRwczovL3d3dy5lZGl0b3JpYWxtYW5hZ2VyLmNvbS9wb25lLw..) to access your manuscript.

Your manuscript can be found in the "Revisions Sent Back to the Author" link under the New Submissions menu. After you have made the changes requested above, please be sure to view and approve the revised PDF after rebuilding the PDF to complete the resubmission process.


Kind regards,

Régis Cohen

MD, PhD
